# Case Report: Multidisciplinary management of primary inferior vena cava leiomyosarcoma: a comprehensive case study

**DOI:** 10.3389/fonc.2023.1190276

**Published:** 2023-11-13

**Authors:** Wuchao Li, Xiaoyong Zhang, Yi Zhang, Rongpin Wang

**Affiliations:** ^1^ Department of Radiology, Guizhou Provincial People’s Hospital, Guiyang, Guizhou, China; ^2^ Department of Hepatobiliary Surgery, Guizhou Provincial People’s Hospital, Guiyang, Guizhou, China

**Keywords:** inferior vena cava, leiomyosarcoma, case report, surgical resection, adjuvant therapy

## Abstract

**Introduction:**

Primary Inferior vena cava (IVC) leiomyosarcoma, a rare malignant tumor, presents unique challenges in diagnosis and treatment due to its rarity and the lack of consensus on surgical and adjuvant therapy approaches.

**Case Report:**

A 39-year-old female patient presented with lower limb swelling and mild fatigue. Contrast-enhanced CT identified a tumor mass within the dilated IVC. Abdominal MRI revealed primary IVC leiomyosarcoma extending into the right hepatic vein. A multidisciplinary consultation established a diagnosis and devised a treatment plan, opting for Ex-vivo Liver Resection and Auto-transplantation (ELRA), tumor resection and IVC reconstruction. Pathological examination confirmed primary IVC leiomyosarcoma. Postoperatively, the patient underwent a comprehensive treatment strategy that included radiochemotherapy, immunotherapy, targeted therapy, and PRaG therapy (PD-1 inhibitor, Radiotherapy, and Granulocyte-macrophage colony-stimulating factor). Despite the tumor’s recurrence and metastasis, the disease progression was partially controlled.

**Conclusion:**

This case report emphasizes the complexities of diagnosing and treating IVC leiomyosarcoma and highlights the potential benefits of employing ELRA, IVC reconstruction, and PRaG therapy. Our study may serve as a valuable reference for future investigations addressing the management of this rare disease.

## Introduction

Primary vascular leiomyosarcoma, a rare malignant tumor, is 2% of all leiomyosarcomas (1). The inferior vena cava (IVC) is the primary location where vascular leiomyosarcomas develop, and these tumors were first described by Perl and Virchow in 1871 (2). The prognosis for IVC leiomyosarcomas is discouraging, as study show that only around 49.4% survive for 5 years and a meager 29.5% for 10 years (3). The main treatment option is complete surgical removal, and additional therapy after the surgery may offer some improvement in the prognosis (4, 5). Nevertheless, the lack of agreement on the most suitable surgical approach and postoperative adjuvant therapy decisions is attributable to limited researches, predominantly consisting of individual case reports and small patient series. This case report examines the usefulness of radiodiagnosis, treatment choices, and experiences in a patient with primary unresectable IVC leiomyosarcoma. It also reviews existing literatures on Ex-vivo Liver Resection and Auto-transplantation (ELRA), tumor resection and IVC reconstruction (6–12). This study seeks to expand our understanding of diagnostic and treatment strategies for primary IVC leiomyosarcoma by examining a specific case and reviewing relevant literature.

## Case report

**Figure 1:**
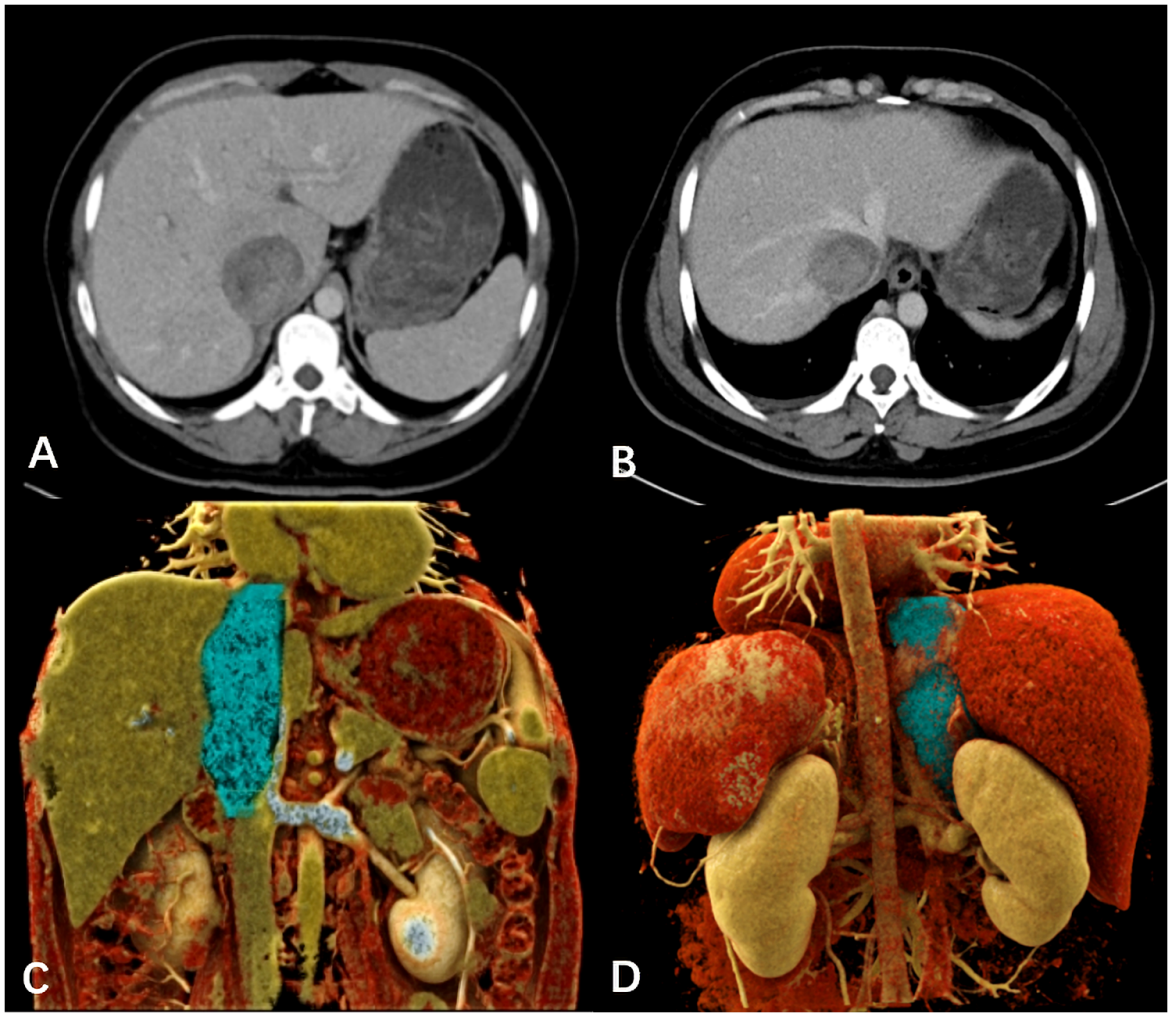
**(A, B)** The venous-phase CT displayed the dilated IVC, seemingly dipping into a slightly enhanced tumor mass invading the right hepatic lobe vein. **(C, D)** The cinematic three-dimensional rendering clearly depicted the tumor size and extent (blue), with the superior margin close to the right atrium entrance and the lower margin at the level of the left renal vein.

**Figure 2:**
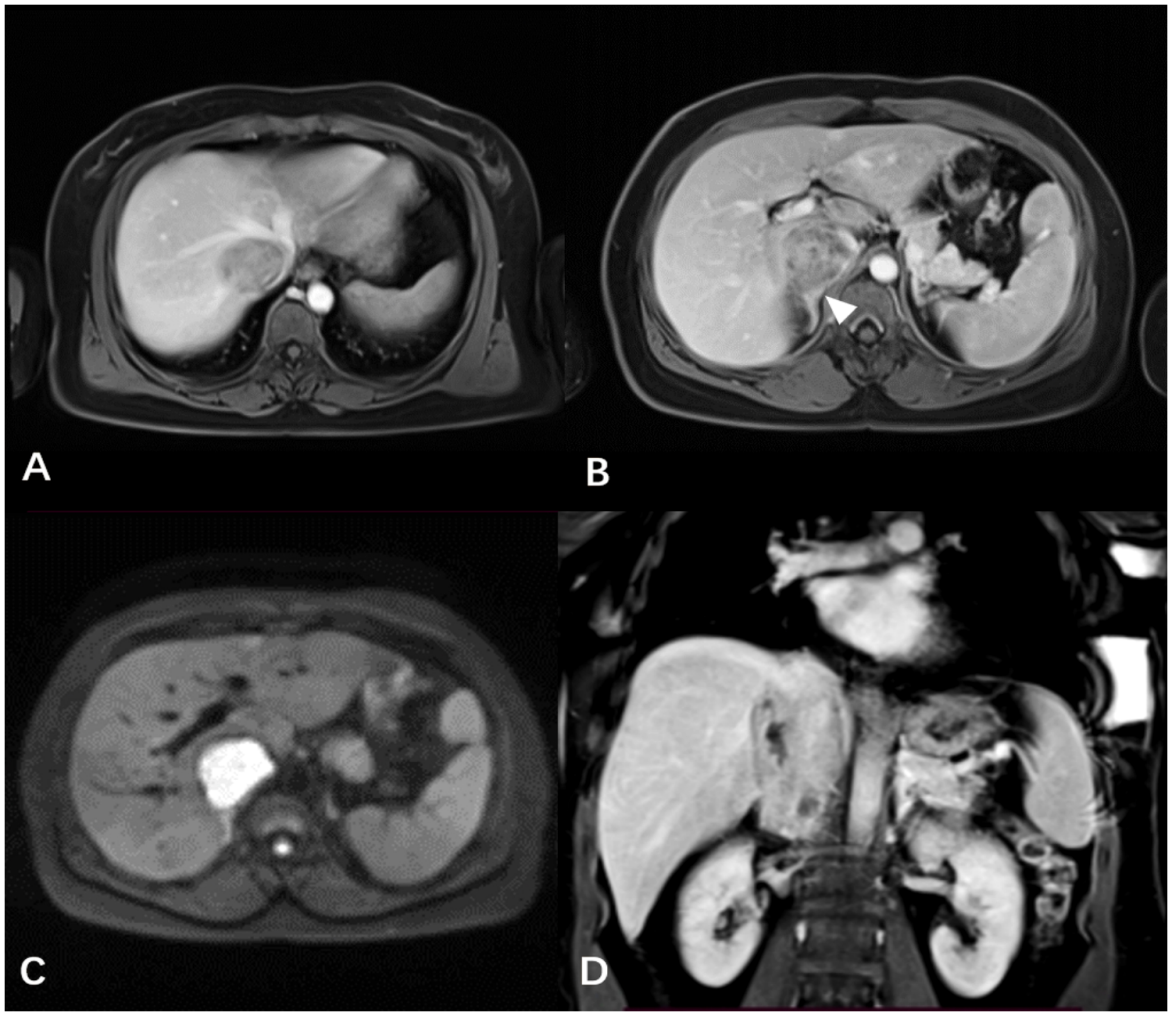
**(A, B)** The contrast-enhanced T1WI revealed the IVC tumor infiltrating the right hepatic lobe vein and vessel wall, along with the indistinct boundary (white arrowheads) between the right adrenal gland and the tumor. **(C)** The axial DWI distinctly showed the IVC tumor with a high signal. **(D)** The mass exhibiting progressive and heterogeneous enhancement was presented using coronal contrast-enhanced T1WI.

A 39-year-old female patient presented with lower limb swelling and mild fatigue persisting for two weeks. Physical examination revealed only lower extremity edema. Her medical records indicate a 20-year history of hepatitis B. Upon conducting liver function tests, abnormalities were observed, with heightened levels of aspartate aminotransferase (180 U/L), alanine aminotransferase (80 U/L), and total bilirubin (14.8 μmol/L). A subsequent contrast-enhanced CT scan of the liver uncovered a tumor mass within the dilated IVC, providing visual evidence in **Figures 1A, B**. Pelvic CT angiography indicated the potential presence of right ovarian vein thrombosis. CT cinematic rendering generated a three-dimensional representation, effectively illustrating the size and extent of the IVC tumor (**Figures 1C, D**). The abdominal MRI examination provided clear evidence of the primary IVC tumor infiltrating the right hepatic vein and breaching the vessel walls, as depicted in **Figure 2**.

In response to this complex scenario, a multidisciplinary consultation was organized to establish an accurate diagnosis and develop a well-rounded treatment approach. Primary malignant IVC tumor diagnosis was reached based on multi-modal imaging after excluding IVC thrombosis or tumor thrombus secondary to liver or kidney tumors. Conventional surgery was deemed unsuitable for tumor resection due to exposure difficulties. Consequently, ELRA, tumor resection, and IVC reconstruction were proposed as alternative treatment options.

**Figure 3:**
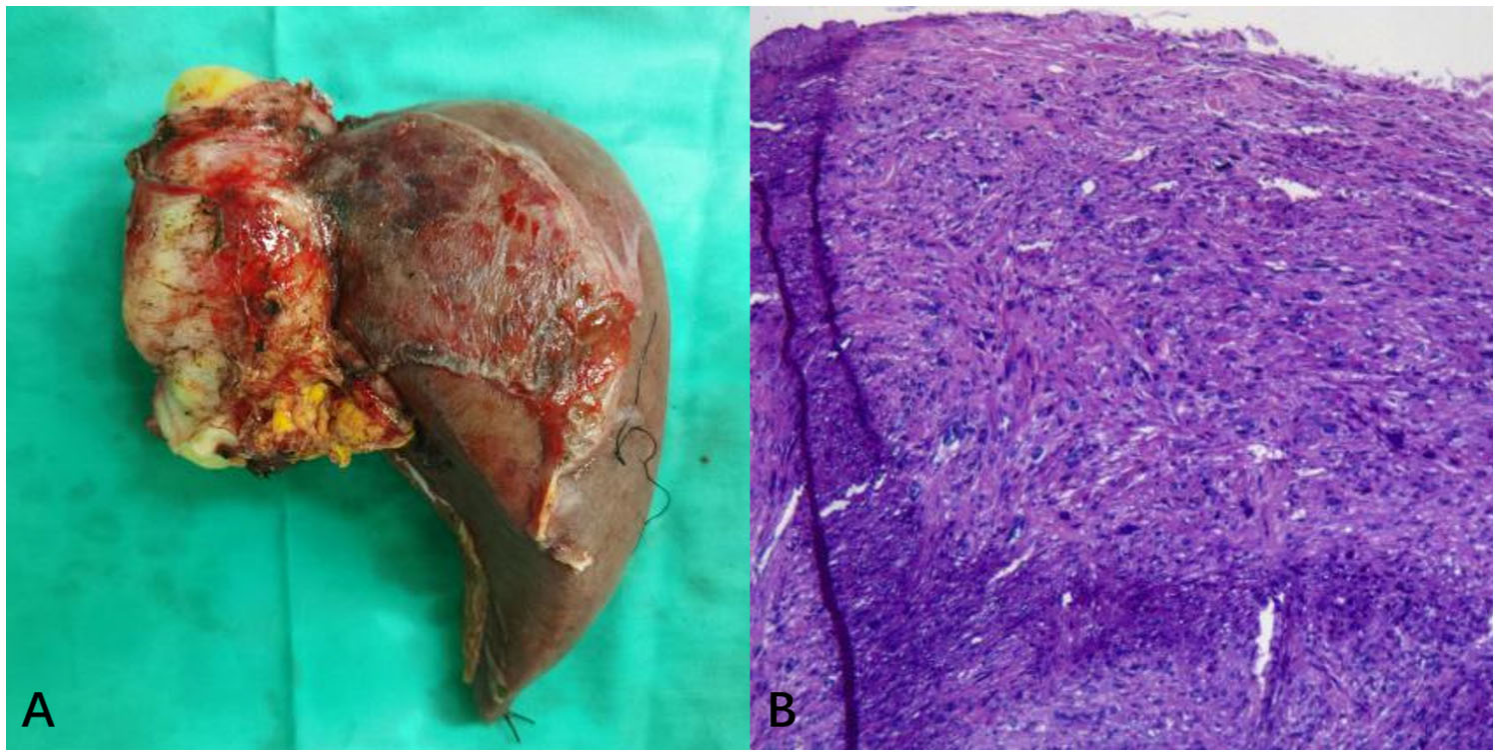
**(A)** The overall appearance of the resected specimen, which consisted of the right liver lobe and IVC tumor. **(B)** H&E stained pathological tissue section. Postoperative pathology demonstrated proliferative spindle-like cells with atypical nuclei (H&E, ×200).

The surgery lasted approximately 11 hours, with an ischemic time of 100 minutes, without the use of a pump. The decision to avoid extracorporeal circulation was made because of our team’s extensive experience, which allowed us to complete the necessary surgical steps within the limited ischemic time while keeping the patient stable. In addition, avoiding extracorporeal circulation reduced potential complications and facilitated the patient’s recovery. The intraoperative blood loss was about 5000 mL, necessitating the administration of 16 units of type B RHD(+) leukocyte-depleted red blood cell suspension, 2200 mL of frozen plasma, and 10 units of cryoprecipitate. Cell saver was not used due to the malignant tumor and potential risk of tumor cell dissemination. Intraoperatively, a firm, immobile mass was identified within the IVC posterior to the liver, extending superiorly to the right atrial entrance and inferiorly to the level of the left renal vein. The entire liver and IVC (thoracic and retrohepatic segments) were resected, followed by autologous left liver transplantation after removing the right liver lobe and tumor. The tumor size measured 8cm × 6cm × 5cm, as depicted in **Figure 3A**. Subsequently, the IVC was reconstructed using artificial vascular grafts. Given the extensive trauma and prolonged surgical duration, the patient was transferred to the ICU for specialized care. Ventilator-assisted breathing was required, and a high fever developed, managed with antibiotic therapy. Following a four-day ICU stay, the patient’s condition stabilized, enabling their relocation to a general hospital ward. The overall hospitalization duration was 15 days. According to the Clavien-Dindo classification, the surgical complication was gradeII. Pathological examination confirmed primary IVC leiomyosarcoma infiltrating all vessel wall layers without invading surrounding tissues, as depicted in **Figure 3B**. Abdominal CT follow-up within one month postoperatively demonstrated patent artificial IVC and left and middle hepatic veins.

Following the operation, the patient first went through adjuvant radiotherapy, then chemotherapy. Doxorubicin (40 mg intravenously guttae (ivgtt), days 1-3) and isocyclophosphamide (3.0 g continuous intravenous infusion (civ), days 1-5 for the first cycle; 5 g civ days 1-2 and 7.5 g civ days 3-5 for the second cycle) were used as the first-choice chemotherapy drugs for two cycles. Because of serious side effects, particularly bone marrow suppression, doxorubicin (30 mg ivgtt, days 1-3) and dacarbazine (300 mg ivgtt, days 1-3) were administered as an alternative for two cycles. Tragically, the patient experienced tumor recurrence and liver metastases 12 months after surgery, leading to a switch in treatment to immunotherapy and targeted therapy. A revised regimen was implemented, featuring tislelizumab (200 mg ivgtt, day 1) and anlotinib (12 mg per os (po), days 1-14) for two 21-day cycles, with anlotinib continued orally thereafter. At the 17-month postoperative stage, newly appeared lung metastases and progressed hepatic lesions were found, leading to an exploratory treatment of the liver and lung metastases with PD-1 inhibitor, Radiotherapy, and Granulocyte-macrophage colony-stimulating factor (PRaG) therapy for one cycle each. Then, continued oral administration of anlotinib was maintained. Four months later, corresponding to the two-year mark post-surgery, the disease did not show significant progression, indicating partial disease control.

## Discussion

Primary IVC leiomyosarcoma is a rare cancer that affects females more than males, with a ratio of 4:1. It typically appears around the age of 54 (13, 14). Early diagnosis is challenging due to its subtle onset, slow growth, and non-specific symptoms (15). The tumor can be put into three segments based on which part of the IVC is involved: SegmentI(below the kidney area, 34%), Segment II (between the kidney and liver area, 42%), and Segment III (from the liver veins to the right upper heart chamber, 24%) (16). In this case, both Segments II and III were involved. Although complete surgical removal is widely recognized as an effective treatment, the tumor’s extensive and deep location presents significant surgical obstacles. Hence, thorough preoperative assessment of tumor characteristics, accurate determination of tumor extent, and careful surgical planning are essential, with imaging techniques playing a critical role.

Contrast-enhanced CT, a prevalent radiological technique, is often utilized for preoperative assessments of IVC leiomyosarcoma (15). CT cinematic rendering, an advanced reconstruction technique, provides enhanced visualization of the tumor location and size, facilitating pre-surgical planning and improving communication between healthcare professionals and patients (17). MRI can provide better soft tissue resolution compared to CT and can clearly show the shape of the tumor, adjacent tissues and surrounding tissue relationships (18). Diffusion-weighted imaging, which doesn’t require contrast agents, excels at visualizing tumors and tumor thrombi due to its ability to detect restricted diffusion caused by increased tumor cell density and nuclear-cytoplasmic ratio. The main radiographic features of IVC leiomyosarcoma include an enlarged IVC with an intraluminal soft-tissue mass exhibiting irregular enhancement. Differential diagnosis primarily involves distinguishing between a secondary tumor thrombus and a clot-derived thrombus. Advanced imaging techniques are crucial for preoperative evaluation, and interdisciplinary collaboration significantly improves IVC leiomyosarcoma management by providing a comprehensive understanding and better treatment strategies.

In this case, the tumor’s extensive involvement and deep location made conventional surgical methods unable to achieve R0 resection. The tumor reached the right atrium’s entrance and infiltrated the right hepatic vein. We chose the ELRA surgical approach based on previous research on IVC leiomyosarcoma (11, 12). ELRA presents several advantages over standard methods, including more precise lesion removal, a reduced risk of uncontrollable bleeding, and fewer postoperative complications (18). Managing the IVC after resection is a critical consideration. The methods of ligation, repair, and reconstruction of the IVC after complete tumor removal remain a debated issue. IVC ligation may reduce the risk of leg edema for lesions located below the hepatic vein level, provided that sufficient collateral circulation has developed. Recent studies suggest that IVC repair or reconstruction may improve postoperative survival rates for most primary leiomyosarcoma patients (19). In this case, due to the adoption of the ELRA approach for curative tumor removal, IVC reconstruction became a necessity.

Postoperatively, the patient demonstrated a patent superior vena cava without thrombosis, notable improvement in leg edema, and favorable recovery. The combination of ELRA, tumor resection and IVC reconstruction presents a feasible surgical option for patients with unresectable IVC leiomyosarcoma, although careful evaluation of the procedure’s complexity and associated risks is essential. To gain a comprehensive understanding of the therapeutic efficacy of combining ELRA and IVC reconstruction, we reviewed and collated clinical, surgical, and postoperative follow-up data from previous cases. These cases involved the utilization of ELRA, tumor resection and IVC reconstruction as the primary intervention for IVC leiomyosarcoma. The organized information has been tabulated in **Table 1** for ease of comparison (6–12).

**Table 1:** Comparative patient characteristics & outcomes: ELRA and IVC reconstruction in IVC leiomyosarcoma.

Reference	Age/ Gender	Symptoms	Size (in cm)	Anatomical Classification of IVC LMS	Surgery method	Extracorporeal Circulation	Anhepatic Phase (minutes)	Operative Time (minutes)	Intraoperative Blood Loss (liters)	Transfusion	Resection Margin	Postoperative Complications	Hospital Stay (days)	Adjuvant Therapy	Reoperation	Recurrence	Time to Recurrence (months)	Current Status	Follow-up Time (months)
Brekke et al. 2003 (6)	64/M	Abdominal discomfort	6×6×7	Segment II	ELRA, tumor resection, IVC reconstruction	Yes (veno-venous bypass)	283	458	Not reported	Yes	Negative	No	11	No therapy	No	No	Not applicable	Alive	24
Takatsuki et al. 2014 (7)	40/F	Leg swelling	Not reported	Segment II–III	ELRA, tumor resection, IVC reconstruction	Yes (artificial cardiopulmonary device)	Not reported	859	15.5	Not reported	Not reported	No	Not reported	No therapy	No	No	Not applicable	Alive	6
Fernandez et al. 2015 (8)	52/F	Leg swelling, breathlessness	15	Segment I–III	ELRA, tumor resection, bilateral nephrectomy, left renal autotransplantation, IVC reconstruction	Yes (venous-venous bypass)	Not reported	Not reported	Not reported	Not reported	Negative	Kidney dysfunction	Not reported	Chemotherapy	No	No	Not applicable	Alive	12
Bunting et al. 2017 (9)	53/F	Abdominal distention, bloating	8.8 × 5 × 4.7	Segment II–III	ELRA, tumor resection, IVC reconstruction, atriocaval reconstruction	Yes (cardiopulmonary bypass)	166	813	6	Yes	Negative	Bilateral pleural effusions	10	No therapy	Yes, pulmonary wedge resection	Yes (pulmonary metastasis)	14	Alive with tumor	14
Buchholz et al. 2019 (10)	58/F	Abdominal collaterals, right subcostal tumor	5.7 × 5.7 × 11	Segment I–II	ELRA, tumor resection, IVC reconstruction	No	120	575	Not reported	Not reported	Negative	Chest infection, kidney dysfunction	Not reported	No therapy	No	Yes (Local recurrence)	12	Alive with tumor	24
Tuxun et al. 2021 (11)	33/F	Abdominal distention, leg swelling	6.5 × 4.5 × 3.5	Segment II–III	ELRA, tumor resection, IVC reconstruction, hepatic vein thrombectomy, atrial reconstruction	Yes (cardiopulmonary bypass)	128	720	1.5	Yes	Negative	Liver dysfunction	21	Radiotherapy	Yes, hepatic radiofrequency ablation	Yes (hepatic recurrence)	24	Alive with tumor	32
Sharma et al. 2022 (12)	67/M	Back and lower abdominal pain	Not reported	Segment II	ELRA, tumor resection, IVC and hepatic vein-atrial reconstruction	Yes (cardiopulmonary bypass)	Not reported	Not reported	Not reported	Yes	Negative	No	5	Radio-Chemotherapy	No	No	Not applicable	Alive	3

From our literatures review and data analysis in **Table 1**, we acknowledge the complexity of ELRA and IVC reconstruction. This method has inherent risks due to tumor involvement, extended surgery times, and notable blood loss. Although not ideal, this approach remains effective for patients with unresectable IVC leiomyosarcoma. It can achieve tumor clearance and manage postoperative complications. Nonetheless, around 50% of people in **Table 1** faced tumor recurrence or metastasis within two years after surgery. Therefore, it is crucial to closely monitor these patients, and personalized follow-up treatments may play a vital role in managing recurrent or spreading cancer.

Postoperative adjuvant chemotherapy and radiotherapy are frequently utilized as auxiliary treatments for IVC leiomyosarcoma (5). Doxorubicin and isocyclophosphamide, serving as cornerstone chemotherapy agents, are employed extensively (5). However, in comparison to the chemotherapy response observed in synovial sarcoma and liposarcoma, inferior response rates of only 10% to 25% are witnessed in IVC leiomyosarcoma (20). Consequently, IVC leiomyosarcoma frequently results in local or distant recurrence. In this case, liver tumor recurrence and metastasis occurred one year after treatment. To control the disease progression, targeted therapy combined with PD-1 inhibitors was administered, as previous studies have suggested their effectiveness (21). Nonetheless, the patient didn’t improve as expected, lung metastases developed four months later, possibly due to the advanced stage of tumor and the large tumor size. Eventually, the patient underwent the PRaG treatment. This therapy stimulates the patient’s immune system to combat tumor cells by combining radiation therapy, a substance called GM-CSF, and PD-1 inhibitors (22, 23). The partial improvement of the patient’s condition after PRaG treatment suggests that this approach can be considered as an alternative where conventional treatment modalities prove to be ineffective or contraindicated.

In summary, this case report emphasizes the challenges in diagnosing and treating primary unresectable IVC leiomyosarcoma. The use of ELRA and IVC reconstruction for radical resection, along with the innovative PRaG therapy for postoperative recurrence and metastasis, offers a potential solution for this rare disease. However, Further studies are necessary to determine optimal and consistent surgical and adjuvant treatment methods for IVC leiomyosarcoma.

## Data availability statement

The raw data supporting the conclusions of this article will be made available by the authors, without undue reservation.

## Ethics statement

The studies involving humans were approved by Ethics Committee of Guizhou Provincial People’s Hospital, Guizhou Province, China. The studies were conducted in accordance with the local legislation and institutional requirements. The human samples used in this study were acquired from a by- product of routine care or industry. Written informed consent for participation was not required from the participants or the participants’ legal guardians/next of kin in accordance with the national legislation and institutional requirements. Written informed consent was obtained from the individual(s) for the publication of any potentially identifiable images or data included in this article.

## Author contributions

The writing of the manuscript was led by WL, with XZ responsible for image creation. The manuscript was subsequently revised by RW and YZ. All authors contributed to the article and approved the submitted version.

## References

[1] Rusu CB Gorbatâi L Szatmari L Koren R Bungărdean CI Feciche BO . Leiomyosarcoma of the inferior vena cava. Our experience and a review of the literature. Romanian J Morphol Embryol (2020) 61(1):227. doi: 10.47162/RJME.61.1.25 PMC7728114 32747914

[2] Perl L Virchow R . Ein Fall von Sarkom der Vena cava inferior. Archiv für pathologische Anatomie und Physiologie und für klinische Medicin. (1871) 53(4):378–83. doi: 10.1007/BF01957198

[3] Saraswathy M . Primary leiomyosarcoma of inferior vena cava-a case report. Univ J Surg Surg Specialities (2021) 7(4).

[4] Pan J Qiu C-Y He Y-Y Xue X Li D-L Tian L . A 10-year experience of leiomyosarcoma of the inferior vena cava. Phlebology (2022) 37(8):572–8. doi: 10.1177/02683555221101706 35570826

[5] Saikia J Rastogi S Barwad A Dhamija E Pandey R Bhoriwal S . A systematic review of the current management approaches in leiomyosarcoma of inferior vena cava—Results from analysis of 118 cases. Asian Cardiovasc Thorac Ann (2022) 30(3):349–63. doi: 10.1177/02184923211049911 34672808

[6] Brekke IB Mathisen Ø Line P-D Hauss HJ . Hepatic autotransplantation with ex situ neoplasm extirpation and vena cava replacement. Hepato-gastroenterology (2003) 50(54):2169–72. 14696489

[7] Takatsuki M Eguchi S Hashizume K Soyama A Hidaka M Tanigawa K . Liver autotransplantation for an inferior vena cava tumor. Transplantation (2014) 98(12):e92–e4. doi: 10.1097/TP.0000000000000516 25955342

[8] Fernandez HT Kim PT Anthony TL Hamman BL Goldstein RM Testa G . Inferior vena cava reconstruction for leiomyosarcoma of zone I–iii requiring complete hepatectomy and bilateral nephrectomy with autotransplantation. J Surg Oncol (2015) 112(5):481–5. doi: 10.1002/jso.24041 26356493

[9] Bunting B Marsh JW Wei L Hughes C Gelzinis TA . Surgical resection of a leiomyosarcoma involving atrial reconstruction, cardiopulmonary bypass, and ex-vivo liver resection and reimplantation. J cardiothoracic Vasc Anesth (2017) 31(2):637–41. doi: 10.1053/j.jvca.2016.06.019 27645825

[10] Buchholz BM Taniere P Isaac J Gourevitch D Muiesan P . Autotransplantation of the liver for ex vivo resection of intrahepatic caval leiomyosarcoma: A case report. Exp Clin Transplantation: Off J Middle East Soc Organ Transplant (2019) 18(3):396–401. doi: 10.6002/ect.2018.0183 30880647

[11] Tuxun T Li T Apaer S He Y-B Bai L Gu S-S . Ex vivo liver resection and autotransplantation as surgical option for zone ii–iii leiomyosarcoma of ivc: A case report and literature review. Front Oncol (2021) 11:690617. doi: 10.3389/fonc.2021.690617 34178689 PMC8226245

[12] Sharma NK Okakpu U Murthy J Wei LM Lopez-Solis R Schmidt C . Case report: surgical resection of a retro-hepatic leiomyosarcoma involving atrial reconstruction, cardiopulmonary bypass, ex vivo tumor resection, and liver re-implantation. Front Surg (2022) 9. doi: 10.3389/fsurg.2022.1037312 PMC9676270 36420407

[13] Nanashima A Takamori H Imamura N Furukawa K Hiyoshi M Hamada T . Successful right hepatectomy for recurrent liver tumor originating from an inferior vena cava leiomyosarcoma: A follow-up case report. Am J Case Rep (2022) 23:e938009–1. doi: 10.12659/AJCR.938009 PMC9623539 36301744

[14] Mingoli A Cavallaro A Sapienza P Di Marzo L Feldhaus R Cavallari N . International registry of inferior vena cava leiomyosarcoma: analysis of a world series on 218 patients. Anticancer Res (1996) 16(5B):3201–5. doi: 10.1006/ijhc.1996.0010 8920790

[15] Li X Li B Zhang N Wang F Zhang C Sun N . Case report: reconstruction of the left renal vein with resected autologous right renal vein interposition after excision of an inferior vena cava leiomyosarcoma. Front Surg (2022) 9. doi: 10.3389/fsurg.2022.913927 PMC9362845 35959128

[16] Ceyhan M Danaci M Elmali M Özmen Z . Leiomyosarcoma of the inferior vena cava. Diagn interventional Radiol (2007) 13(3):140. 17846988

[17] Javed AA Young RW Habib JR Kinny-Köster B Cohen SM Fishman EK . Cinematic rendering: novel tool for improving pancreatic cancer surgical planning. Curr problems Diagn Radiol (2022) 541(6):878–83. doi: 10.1067/j.cpradiol.2022.04.001 35595587

[18] Zhou X Wang M Li S Cai H Liang L Li Z-P . A case of a huge inferior vena cava leiomyosarcoma: precise preoperative evaluation with gadobutrol-enhanced mri. Cancer Manage Res (2020) 12:7929. doi: 10.2147/CMAR.S258990 PMC7473983 32943927

[19] Gaignard E Bergeat D Robin F Corbiere L Rayar M Meunier B . Inferior vena cava leiomyosarcoma: what method of reconstruction for which type of resection? World Journal of Surgery (2020) 44:3537–44. doi: 10.1007/s00268-020-05602-2 32445073

[20] Krikelis D Judson I . Role of chemotherapy in the management of soft tissue sarcomas. Expert Rev Anticancer Ther (2010) 10(2):249–60. doi: 10.1586/era.09.176 20132000

[21] Habra MA Stephen B Campbell M Hess K Tapia C Xu M . Phase ii clinical trial of pembrolizumab efficacy and safety in advanced adrenocortical carcinoma. J immunothe Cancer (2019) 7:1–9. doi: 10.1186/s40425-019-0722-x PMC6751592 31533818

[22] Kong Y Zhao X Xu M Pan J Ma Y Zou L . Pd-1 inhibitor combined with radiotherapy and gm-csf (Prag) in patients with metastatic solid tumors: an open-label phase ii study. Front Immunol (2022) 13. doi: 10.3389/fimmu.2022.952066 PMC9304897 35874780

[23] Xu H Hong Z Xu M Kong Y Ma Y Shan C . Prag therapy of refractory metastatic gastric cancer: A case report. Front Immunol (2022) 13. doi: 10.3389/fimmu.2022.926740 PMC9300850 35874658

